# Mr. William Henry Folker

**Published:** 1912-05-25

**Authors:** 


### Obituary.

Mr. William Henry Folker, consulting surgeon to the North Staffordshire Infirmary, has died at Hanley at the age of eighty-six. It was in 1853 that Mr. Folker became first connected with the North Staffordshire Infirmary—a connection which, through one office or another, had lasted up to the time of his decease. His first office was that of house surgeon, followed by election as honorary surgeon, and later as ophthalmic surgeon, an office which, it is interesting to note, is now held by his son. Mr. Folker, after joining the consulting staff of the infirmary, was elected in turn vice-president and president, the latter in 1906. He became qualified in 1851 after studying at Charing Cross Hospital and in Paris.

